# Rheb and mammalian target of rapamycin in mitochondrial homoeostasis

**DOI:** 10.1098/rsob.130185

**Published:** 2013-12

**Authors:** Marlous J. Groenewoud, Fried J. T. Zwartkruis

**Affiliations:** Molecular Cancer Research, Centre for Biomedical Genetics and Cancer Genomics Centre, University Medical Center Utrecht, Universiteitsweg 100, 3584 CG Utrecht, The Netherlands

**Keywords:** mammalian target of rapamycin, mitochondria, metabolism, Rheb, mitophagy

## Abstract

Mitochondrial dysfunction has been associated with various diseases, such as cancer, myopathies, neurodegeneration and obesity. Mitochondrial homoeostasis is achieved by mechanisms that adapt the number of mitochondria to that required for energy production and for the supply of metabolic intermediates necessary to sustain cell growth. Simultaneously, mitochondrial quality control mechanisms are in place to remove malfunctioning mitochondria. In the cytoplasm, the protein complex mTORC1 couples growth-promoting signals with anabolic processes, in which mitochondria play an essential role. Here, we review the involvement of mTORC1 and Rheb in mitochondrial homoeostasis. The regulatory processes downstream of mTORC1 affect the glycolytic flux and the rate of mitophagy, and include regulation of the transcription factors HIF1α and YY1/PGC-1α. We also discuss how mitochondrial function feeds back on mTORC1 via reactive oxygen species signalling to adapt metabolic processes, and highlight how mTORC1 signalling is integrated with the unfolded protein response in mitochondria, which in *Caenorhabditis elegans* is mediated via transcription factors such as DVE-1/UBL-5 and ATFS-1.

## 2. Introduction

Metabolic processes in a cell require the continuous input of energy in the form of ATP. In the presence of nutrients and oxygen, mitochondria are the major suppliers of ATP. The metabolic process inside mitochondria that uses energy released during the oxidation of nutrients to produce ATP is called oxidative phosphorylation (OXPHOS). Depending on the cell type, mitochondria use derivatives of carbohydrates, fats or amino acids to fuel the tricarboxylic acid (TCA) cycle. When glucose is used as the primary energy source, it is processed into two molecules of pyruvate via the glycolytic pathway. These will enter the mitochondrial matrix (MM) where they will be converted to acetyl-CoA. Oxidation of this metabolite generates electrons for transport along the respiratory chain in the inner mitochondrial membrane (IMM). This transport of electrons results in the pumping of H^+^ from the MM to the intermembrane space (IMS), generating a pH gradient and a membrane potential (Δ*ψ*_m_) across the IMM. Energy from H^+^ ions that flow down the electrochemical gradient is used by the ATP synthase for the conversion of ADP and P_i_ to ATP. As a by-product of OXPHOS, reactive oxygen species (ROS) are produced in the mitochondria. ROS produced in low levels have a signalling function in the cell, but high levels are damaging to mitochondria. Damaged mitochondria produce excess ROS and this leads to a vicious cycle of more damaged mitochondria with concomitant ROS production. Expression of uncoupling proteins allows H^+^ ions to cross the IMM without production of ATP in order to generate heat or lower the Δ*ψ*_m_ to prevent ROS formation. Apart from the generation of ATP, the electrochemical gradient is also used for import of proteins and metabolites that are used to sustain the function of the mitochondria themselves. The function of mitochondria also includes the formation of building blocks for amino acid and fatty acid synthesis, storage of Ca^2+^ and regulation of apoptosis (reviewed in [1]). Obviously, because of the central role of ATP in metabolic processes and the damaging effects of excess ROS there must be a strict coupling between the import of energy sources into the cell, metabolic pathways and the functionality of mitochondria.

Mitochondrial homoeostasis depends on control mechanisms to regulate the number of mitochondria as well as their quality. Cells respond to an increase in metabolic demand by increasing their number of mitochondria (reviewed in [2,3]). Under conditions of low metabolic activity an excess of mitochondria can selectively be removed via a specialized form of autophagy, named mitophagy (reviewed in [4]). Quality control of mitochondria is ensured via various mechanisms. First, mitochondria undergo continuous cycles of fusion and fission that may support their function by preventing stochastic loss of metabolic substrates or mitochondrial DNA (reviewed in [5]). Second, when stress situations, such as mutation of mitochondrial DNA or excessive ROS production induced by physiological stimuli, result in the presence of damaged or improperly folded proteins, a mitochondrial unfolded protein response (UPR^mt^) is triggered. This response leads to transcriptional activation of mitochondrial chaperone proteins that reduce the number of misfolded proteins (reviewed in [6]). Finally, quality of mitochondria is also maintained via the mitophagic pathway, which prevents the production of excess ROS. Ultimately, if mitochondrial damage becomes too pervasive, the cell undergoes apoptosis by activation of caspases, triggered by the release of cytochrome *c* from the IMS [7]. As the vast majority of mitochondrial proteins are encoded by the nuclear genome, signalling pathways are required to regulate nuclear transcription of mitochondrial genes such that optimal mitochondrial function ensues. An important signalling pathway in this regulation is the target of rapamycin (TOR) signalling pathway. The kinase TOR is a critical player in the tight coupling of cellular metabolism and mitochondria in various organisms (reviewed in [8,9]). The scope here is to review the role of mammalian TOR (mTOR) and discuss the various modes by which mTOR influences these organelles.

## 3. Mammalian target of rapamycin signalling pathway

**Figure 1.**
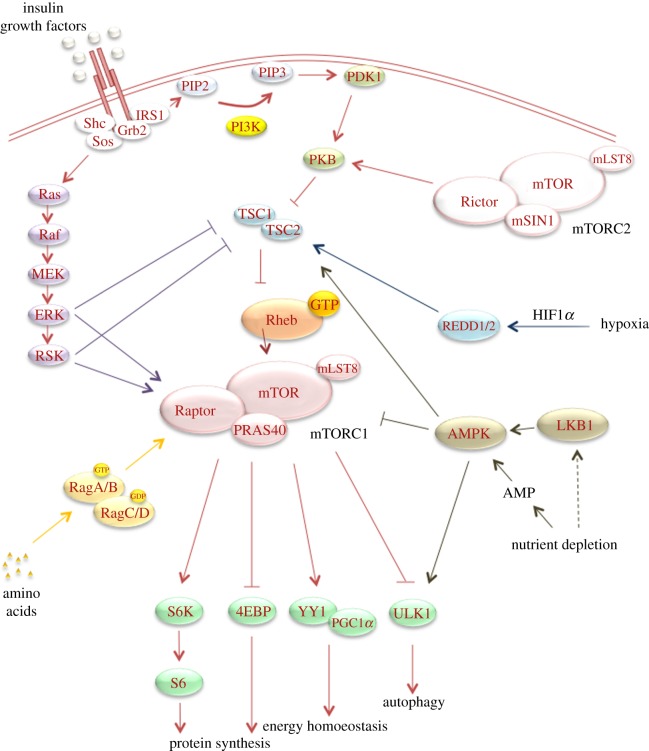
mTOR signalling pathway. Overview of major upstream regulatory signals of mTORC1 and major downstream effectors of mTORC1. Insulin and growth factors stimulate mTORC1 activity by binding growth factor receptors and stimulating downstream signalling. Activation leads to PIP3 generation by PI3K. PIP3 recruits PDK1, which in turn activates PKB. PKB represses the GAP activity of the TSC1/2 complex towards Rheb, which leads to GTP-loaded active Rheb and subsequent activation of mTORC1. Growth factors can simultaneously activate MAPK signalling, which stimulates mTORC1 activity via the inactivation of the TSC1/2 complex and the direct phosphorylation of Raptor. Finally, growth factors stimulate mTORC2 activity in a PI3K-dependent manner, but by an unknown mechanism. Amino acids also stimulate mTORC1 activity by activating the Rag GTPases. This activation recruits mTORC1 to the lysosomes where it is in close proximity to Rheb. Negative regulation of mTORC1 activity occurs when cells are deprived of oxygen or nutrients. Reduced availability of oxygen stabilizes HIF1α and thereby activates the REDD1/2 proteins that stimulate the TSC1/2 complex. Nutrient depletion stimulates the TSC1/2 complex via the activation of AMPK via either LKB1 activation or an increase in AMP levels. In addition, AMPK directly inhibits mTORC1 activity via the phosphorylation of Raptor. Upon activation, mTORC1 regulates a subset of downstream effects such as protein synthesis via S6K and 4EBP, autophagy via Ulk1 phosphorylation and energy homoeostasis via PGC-1α and YY1.

TOR is a highly conserved serine–threonine kinase that plays an important role in cell growth, autophagy and metabolism in response to growth factors, nutrients, hypoxia and energy stress (figure 1). In mammalian cells, the kinase exists in two complexes. Complex 1 (mTORC1) consists of mTOR, Raptor, mLST8/GβL and PRAS40. This complex integrates growth factor signalling with the availability of nutrients and can be selectively inhibited by the fungicidal macrolide rapamycin. The second complex (mTORC2) consists of mTOR, Rictor, Sin1 and mLST8/GβL and is largely insensitive to nutrients and rapamycin (reviewed in [10]).

### 3.1. Mammalian target of rapamycin complex 1 regulation by growth factors

Growth factors signal to mTORC1 through the phosphoinositide 3-kinase/protein kinase B (PI3K/PKB) pathway and the Ras/mitogen-activated protein kinase (MAPK) pathway. For example, activation of insulin and insulin-like growth factor 1 (IGF-1) receptors induces binding and phosphorylation of adapter proteins such as insulin receptor substrate 1 (IRS1), which then transmits the signal to the PI3K and the MAPK pathways. For the PI3K pathway, phosphorylation of phosphatidylinositol-(4,5)-biphosphate (PtdIns(4,5)P_2_; PIP2) by activated PI3K leads to the generation of PtdIns(3,4,5)P_3_ (PIP3) at the plasma membrane and the subsequent activation of phosphoinositide-dependent kinase-1 (PDK1) [11,12]. PtdIns(3,4,5)P_3_ also recruits PKB to the plasma membrane (reviewed in [13]) and following activation by PDK1, PKB phosphorylates and inactivates the tuberous sclerosis complex 1/2 (TSC1/TSC2) complex [14]. TSC2 harbours a GTPase-activating protein (GAP) domain in its C-terminal region, which stimulates the GTPase activity of the small GTPase Rheb [15–19]. When TSC2 is inhibited, the resulting increase in GTP-bound Rheb will activate mTORC1. In turn, mTORC1 phosphorylates and activates its downstream substrate ribosomal protein S6 kinase 1 (S6K1), leading to 5′ TOP mRNA translation and cell growth [20]. S6K1 also phosphorylates and inactivates IRS1, thereby providing a negative feedback loop [21]. Phosphorylation of the other well-studied substrate of mTORC1, 4EBP1, releases it from the eukaryotic initiation factor eIF4E, thereby allowing eIF4E to initiate cap-dependent translation and to stimulate proliferation [20,22].

The Ras/MAPK pathway is triggered via translocation of the adapter protein Grb2 with SOS, a Ras-specific guanine nucleotide exchange factor (GEF) that associates with Grb2, to the membrane. In the case of insulin/IGF receptor signalling, this involves IRS1. Once activated, MAPK will enhance p90 ribosomal S6 kinase. Both kinases have a dual role in the activation of mTORC1. First, they have both been reported to phosphorylate and inactivate TSC2 [23,24]. Secondly, they phosphorylate the mTORC1 complex protein Raptor on distinct sites [25,26].

### 3.2. Mammalian target of rapamycin complex 1 regulation by nutrients

As alluded to above, the mTORC1 pathway integrates growth factor signalling with nutritional status. It is important to note here that the negative regulation of mTORC1 caused by the lack of nutrients is dominant over positive inputs coming from growth factors. Remarkably, nutrient deprivation does not affect signalling components upstream of mTORC1, such as the insulin receptor or PKB. Multiple pathways have been delineated that serve to signal a shortage of one or more nutrients (reviewed in [27]). A lack of amino acids leads to a block of mTORC1 via a mechanism that is distinct from that arising from an insufficiency in carbohydrates [28]. Furthermore, various pathways often cooperate to fine-tune the response to a given type of nutrient. A major mechanism via which amino acid availability results in mTORC1 activity involves the heterodimeric Rag GTPases (RagAB/CD) [29]. The Rag GTPases are localized on lysosomes/Rab7 positive vesicles via their interaction with the so-called Ragulator complex (p18–p14–MP1–HBXIP–C7Orf59 complex), which has GEF activity towards RagA/B [30,31]. If cells are starved of amino acids, RagA/B is in its GDP-bound state and RagC/D is in its GTP-bound state, which results in cytoplasmic localization of mTORC1. When cells are replenished with amino acids, RagC/D becomes GDP-bound and RagA/B becomes GTP-bound. This will target mTOR to lysosomes via the GTP-dependent interaction of RagA/B with Raptor. The co-localization with Rheb, which also resides at lysosomes, then results in mTORC1 activation. It should be kept in mind that other proteins, such as VPS34 and MAP4K3, are also stimulated by amino acids and required for mTORC1 activity [32–35].

The AMP-activated protein kinase (AMPK) signals carbohydrate insufficiency to mTORC1. Glucose deprivation, interference in glycolysis by addition of 2-deoxy-D-glucose or mitochondrial inhibition with e.g. rotenone, all decrease mTORC1 activity by lowering the ATP concentration in a cell [28,36]. A decline in cellular ATP levels and associated rise in cellular AMP levels lead to the activation of AMPK. Activated AMPK stimulates catabolic processes, such as the uptake and metabolism of glucose and fatty acids, and inhibits anabolic processes, such as the synthesis of fatty acids, glycogen, cholesterol and protein synthesis (reviewed in [37,38]). The activated kinase inhibits these processes by phosphorylating multiple target proteins. Among these is TSC2, whose phosphorylation results in lower Rheb-GTP levels, and consequently suppression of mTORC1 activity [39–41]. The observation that cells lacking TSC2 remain partially sensitive to energy stress suggested that AMPK has additional substrates in the mTORC1 pathway. Indeed, the mTORC1 complex member Raptor has been identified as another AMPK substrate [42]. AMPK directly phosphorylates Raptor in an LKB1-dependent manner leading to the binding of 14-3-3 proteins to Raptor and subsequent inhibition of mTORC1. A number of studies report that interfering in mitochondrial activity leads to a tighter interaction of Raptor and mTOR and a decrease in mTORC1 activity [43,44]. Although the precise mechanism behind this interaction is not resolved, it may reflect an effect of AMPK-mediated Raptor phosphorylation and subsequent conformational change in the complex.

Inhibition of mTORC1 lowers protein translation, which is a major energy-consuming process in the cell. Thus, AMPK lowers the ATP demand in cells via the inhibition of mTORC1. If the energy balance is not restored, energy stress will lead to the induction of autophagy. This process, in which cellular components are included in double-membrane vesicles (autophagosomes), which subsequently fuse with lysosomes, serves to generate sufficient metabolites. Here, activation of AMPK and inhibition of mTOR act in concert to stimulate the autophagy initiating kinase Ulk1 (reviewed in [45,46]). When nutrients are sufficient, mTORC1 phosphorylates Ulk1 and thereby prevents its interaction with AMPK. However, a decrease in mTORC1 activity upon starvation allows AMPK to interact with Ulk1 and stimulate it by phosphorylation. Under energy stress both Ulk1 and AMPK negative cells are impaired in their autophagy response. The importance of this response is seen in cells that are devoid of Ulk1 or contain Ulk1 mutant protein that cannot be phosphorylated by AMPK. These cells are more susceptible to apoptosis upon nutrient deprivation [47]. A similar effect is seen when TSC2−/− cells are deprived of glucose [36]. These cells are highly dependent on glucose for their survival. However, mTORC1 inhibition with rapamycin during glucose deprivation prolongs survival of these cells through an OXPHOS-dependent mechanism. This requires that glutamine is present as an energy source, which is converted to glutamate via glutamine dehydrogenase to fuel the TCA cycle. Thus, rapamycin limits ATP usage to a level that can be sustained. In summary, AMPK and mTORC1 are sensors of the energy status of a cell and together form a switch that has control over the use of anabolic versus catabolic processes in a cell.

### 3.3. Mammalian target of rapamycin complex 1 regulation by oxygen levels

Hypoxia leads to a change in the rate of metabolism in order to decrease ATP consumption and subsequently reduce the cellular oxygen demand to maintain metabolic homoeostasis. Hypoxia regulates mTORC1 activity via three different mechanisms. First, hypoxia leads to a decline in ATP levels and accumulation of AMP. This inhibits mTORC1 signalling via AMPK-induced activation of TSC2 and phosphorylation of Raptor as discussed above. Second, the mitochondrial pro-apoptotic proteins BNIP3 and BNIP3L (also known as Nix) are involved in the rapid inhibitory effect of hypoxia on mTORC1. BNIP3 and BNIP3L interact with Rheb and decrease GTP levels on Rheb, thereby inactivating mTORC1 [48]. Finally, under hypoxic conditions prolyl hydroxylation of hypoxia-inducible factor (HIF)1α by the prolyl hydroxylase domain proteins (PHD1–3) is inhibited. This prevents the marking of HIF1α for degradation by the von Hippel–Lindau tumour suppressor protein and leads to stabilization of HIF1α. The induction of HIF1α levels in cells exposed to hypoxic conditions is mTORC1-dependent and can be reversed by treatment of the cells with rapamycin [49,50]. Stabilized HIF1α binds to the constitutively expressed HIF1β to form an active HIF transcription complex, which induces the expression of REDD1 and REDD2. It has been shown that REDD1 and 14-3-3 proteins compete to bind to TSC2. Relieving the inhibitory action of 14-3-3 proteins on TSC2 leads to the stabilization of the TSC complex and subsequent inhibition of mTORC1 activity. This decrease in mTORC1 activity generates a negative feedback response by inducing the degradation of HIF1α resulting in the normalization of HIF1α levels in order to stop the acute response to hypoxia. REDD1 can inhibit mTORC1 signalling even in the presence of constitutive active PKB, indicating that the response of cells to hypoxic conditions overrides the response to mitogenic stimuli [51,52].

## 4. Mitochondria as a target for mammalian target of rapamycin complex 1 signalling

mTORC1 increases cellular mass via its effect on multiple processes such as protein translation, ribosome biogenesis and mitochondrial biogenesis. With respect to mitochondrial function, mTORC1 appears to play a role at multiple levels. Processes that involve mTORC1 are mitochondrial biogenesis, direct regulation of mitochondrial proteins, regulation of uptake and utilization of carbohydrates and regulation of mitophagy. We discuss these processes in detail below.

### 4.1. Mammalian target of rapamycin complex 1 activates transcription of genes for mitochondrial biogenesis

mTORC1 activity is important for stimulation of transcription of genes involved in mitochondrial biogenesis. One of the master regulators controlling these genes is the peroxisome-proliferator-activated receptor coactivator-1α (PGC-1α) [53]. Long-term inhibition of mTORC1 with rapamycin was found to decrease PGC-1α-mediated gene transcription in muscle cells *in vitro*. As a result, mitochondrial DNA content and oxygen consumption were lowered. An opposite effect was seen in TSC2−/− cells, where mTORC1 activity is constitutively high. mTORC1 appears to mediate its effect via the yin-yang 1 (YY1) transcription factor. YY1 can associate with the scaffolding protein Raptor in mTORC1 and is a direct substrate for mTOR. Phosphorylation by mTORC1 enhances the interaction between YY1 and PGC-1α, which stimulates the association of PGC-1α with mitochondrial genes [54]. In line with this finding, muscle-specific deletion of YY1 in mice results in decreased mitochondrial protein content, decreased OXPHOS and eventual exercise intolerance [55]. Overexpression of PGC-1α in muscle appears to be sufficient to increase OXPHOS capacity and leads to improved insulin sensitivity during ageing, further demonstrating the importance of the PGC-1α/YY1 complex for mitochondrial function [56]. The stimulatory effect of mTORC1 on PGC-1α/YY1-induced transcription is also evident following muscle-specific deletion of mTOR [57] or Raptor [58]. In both cases, progressive muscular dystrophy is seen with altered mitochondrial morphology and a decrease in oxidative capacity.

Although the studies above hint at a relatively simple and linear pathway from mTOR via PGC-1α/YY1 towards mitochondrial biogenesis, other studies demonstrate tissue-specific effects and more complex interactions between components of the mTORC1 pathway and mitochondrial biogenesis (table 1). For example, deletion of Raptor in mature adipocytes results in lean mice with a reduction in size and number of adipocytes, but little or no effect on the mass of other tissues [61]. The leanness is most likely to result from increased expression of the mitochondrial uncoupling protein UCP1 in white adipose tissue (WAT), rather than from reduced food intake, lipolysis or physical activity. These mice are protected from diet-induced obesity and maintain better insulin sensitivity. In WAT, as well as in muscle, PKB signalling is enhanced, demonstrating non-cell autonomous effects. Increased PKB activity is most likely due to the disruption of the negative feedback loop from S6K1 to IRS1. Furthermore, while studies in cell lines indicate that the mTORC1 target S6K1 was not involved in PGC-1α/YY1-induced mitochondrial gene transcription [44,54], studies in mice show that whole-body deletion of S6K1 causes a profound increase in the mitochondrial content of skeletal muscle and adipocytes. This is accompanied by increased expression of PGC-1α and mitochondrial genes including those for uncoupling proteins (UCP1 and UCP3) [62]. Mice lacking S6K1 are protected from high-fat diet-induced obesity, which is at least partially explained by increased OXPHOS of triglycerides. Remarkably, loss of S6K1 in skeletal muscle or other tissues results in energy stress as evident from increased levels of AMP and increased AMPK activity [59]. Under energy stress, perhaps induced by high levels of UCP1, increased AMPK rather than mTORC1 activity appears to promote the increase in mitochondrial mass and elevated PGC-1α expression in S6K1-deficient skeletal muscle [60].

**Table 1.** Overview of effects of mTORC1 and downstream effectors on mitochondrial biogenesis. For each study, the cell/tissue type, the target and the method used to interfere with the target are summarized as well as the effects observed. MEFs, mouse embryonic fibroblasts.

cells	target	method	effect	references
(*a*) *in vitro* studies				
MEFs	TSC2	knockout	enhanced interaction YY1 and PGC-1α	[54]
			increased oxygen consumption	
			increased mitochondrial DNA content	
muscle cells	mTORC1	rapamycin	decreased PGC-1α-mediated gene transcription	[54]
			decreased oxygen consumption	
			decreased mitochondrial DNA content	
	YY1	knockdown	decreased mitochondrial gene expression	
			decreased oxygen consumption	
			decreased mitochondrial DNA content	
Jurkat/HEK293	mTORC1	rapamycin	decreased oxygen consumption	[44]
			decreased mitochondrial membrane potential	
	Raptor	knockdown	decreased oxygen consumption	
			decreased mitochondrial membrane potential	
	YY1	overexpression	increased mitochondrial gene expression	[54]
tissue	target	method	effect	references
(*b*) *in vivo* studies				
muscle	YY1	deletion	decreased OXPHOS	[55]
			decreased mitochondrial protein content	
			exercise intolerance	
	PGC-1α	overexpression	increased OXPHOS	[56]
			improved insulin sensitivity	
	mTOR	deletion	decreased oxidative capacity	[57]
			altered mitochondrial morphology	
			muscular dystrophy	
	Raptor	deletion	decreased oxidative capacity	[58]
			altered mitochondrial morphology	
			muscular dystrophy	
	S6K1	deletion	AMPK activation	[59]
			increased AMP/ATP ratio	
			energy stress	
	AMPK	deletion	decreased expression PGC-1α	[60]
			decreased mitochondrial biogenesis	
adipocytes	Raptor	deletion	reduction size and number adipocytes	[61]
			lean mice	
whole body	S6K1	deletion	increased mitochondrial content skeletal muscle	[62]
			increased mitochondrial content adipocytes	
			reduction size and number adipocytes	
			lean mice	

### 4.2. Mammalian target of rapamycin complex 1 regulates mitochondrial activity via phosphorylation of mitochondrial proteins

The second level at which mTORC1 may affect mitochondrial function is via direct modification of mitochondrial proteins. This hypothesis is supported by findings that mTORC1 can associate with mitochondria and by studies with rapamycin [44,63,64]. Using a cell sorting approach, Schieke *et al.* [44] found a positive correlation between mTORC1 activity, Δ*ψ*_m_, maximal oxidative capacity and cellular ATP levels in Jurkat cells. Blocking mTORC1 by a 12-h rapamycin treatment lowers Δ*ψ*_m_ and maximal oxidative capacity without a measurable effect on mitochondrial mass. Interestingly, phosphorylation of many mitochondrial proteins is diminished, but the functional significance of this has not been established. Ramanathan & Schreiber [64] used a shorter rapamycin treatment to exclude transcriptional effects. They found that rapamycin lowers mitochondrial respiration and induces a shift from OXPHOS towards glycolysis as measured by metabolic profiling. They propose that this occurs through a mechanism that involves the interaction of mTOR with the voltage-dependent anion-selective channel (VDAC1) and the anti-apoptotic mitochondrial transmembrane protein Bcl-X_L_. VDAC1 is located in the OMM where it regulates the Δ*ψ*_m_ as a component of the mitochondrial permeability transition pore. Via phosphorylation by mTOR, Bcl-X_L_ may stimulate the permeability of VDAC1 for TCA substrates. Strikingly, the effect of rapamycin can be mimicked with a Bcl inhibitor [64]. However, regulation of VDAC1 is complex and also PKB has been shown to regulate this channel in a glucose- and hexokinase-dependent manner [65].

### 4.3. Mammalian target of rapamycin complex 1 is involved in balancing glycolytic flux with mitochondrial respiration

To sustain cell growth, mTORC1 affects mitochondrial function via regulation of uptake and utilization of carbohydrates. As part of the PI3K/PKB signalling network, mTORC1 can stimulate glucose uptake via increasing the presence and/or activity of the glucose transporter Glut1 on the plasma membrane [66–68]. Importantly, proliferating cells use glucose not only for ATP production but also to provide intermediates for the synthesis of lipids and nucleic acids. To this end, rapidly proliferating cells exhibit enhanced aerobic glycolysis and reduced OXPHOS, a phenomenon that in tumour cells is known as the Warburg effect (reviewed in [69]). This means that cells prevent the conversion of pyruvate derived from glycolysis into acetyl-CoA in mitochondria. Using microarray analysis and metabolomics approaches, mTORC1 has been shown to increase glycolytic flux via HIF1α-mediated transcription of glycolytic genes [67]. Among these upregulated genes is pyruvate dehydrogenase kinase 1 (PDHK1), which phosphorylates mitochondrial pyruvate dehydrogenase and thereby inactivates the pyruvate dehydrogenase complex. As a consequence, pyruvate is metabolized to lactate instead of acetyl-CoA. Other mechanisms such as phosphorylation of PDHK1 and pyruvate kinase-M2 (PKM2) and hydroxylation of PKM2 [70] also contribute to the shunting of pyruvate into lactate [71,72]. The increased glycolytic flux arising from increased glucose uptake and/or increased expression of glycolytic genes may have a feed-forward effect on mTORC1 activity. It has been reported that the glycolytic enzyme glyceraldehyde-3-phosphate dehydrogenase (GAPDH) can bind Rheb and thereby sequester Rheb away from mTORC1 [73–75]. When the glycolytic flux is high, increased levels of glyceraldehyde-3-phosphate will release Rheb from GAPDH, thus activating mTORC1. During tumorigenesis, activation of PI3K/mTORC1 signalling by activation of oncogenes or loss of tumour suppressor genes can also lead to induction of aerobic glycolysis. As a consequence, tumorigenic cells can survive better during phases of insufficient oxygen supply that they are likely to experience in developing tumours and during which glycolysis is further stimulated via stabilization of HIF1α (reviewed in [76]).

Owing to the export of citrate from mitochondria for fatty acid synthesis, cells under the Warburg effect need to replenish their TCA intermediates (anaplerosis). Glutamine is a major source for this in proliferating cells. This occurs via glutaminolysis to form α-ketoglutarate, which after conversion to oxaloacetate is used to restore citrate levels. Intriguingly, the process of glutaminolysis activates mTORC1 via the Rag GTPases and may explain the addiction of tumour cells to glutamine [77,78].

### 4.4. Mammalian target of rapamycin complex 1 and mitophagy

**Figure 2.**
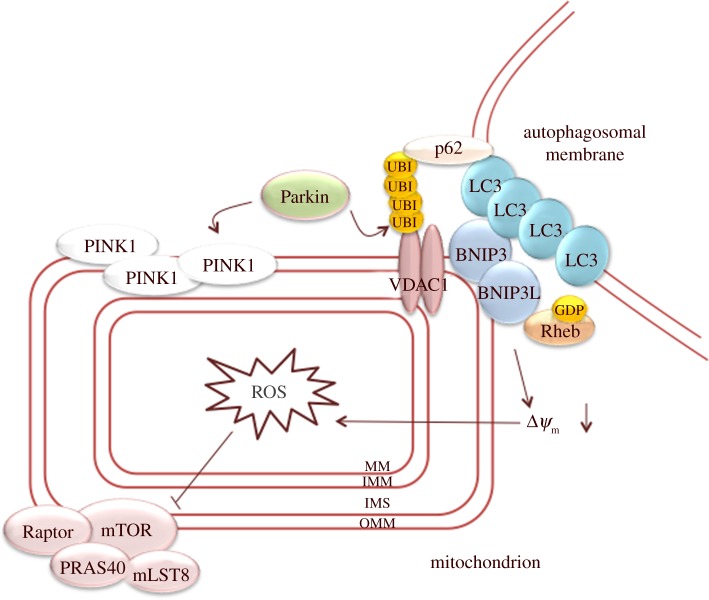
Mitochondrial priming in mitophagy. PINK1 accumulation on the outer mitochondrial membrane recruits Parkin to the mitochondria. In addition, BNIP3 and BNIP3L on the OMM also recruit Parkin to the mitochondria. In turn, Parkin will ubiquitinate mitochondrial proteins, such as VDAC1. Ubiquitinated proteins are recognized by p62, which interacts with LC3 on autophagosomes. In addition, BNIP3 and BNIP3L also interact with the autophagosomal protein LC3, thereby inducing association with the autophagosomal membrane. The increase in ROS production induced by BNIP3 and BNIP3L via the depolarization of Δ*Ψ*_m_ will inhibit mTORC1 activity. In addition, the inactivation of Rheb by BNIP3 and BNIP3L will inhibit mTORC1 activity. MM, mitochondrial matrix; IMM, inner mitochondrial membrane; IMS, intermembrane space; OMM, outer mitochondrial membrane; ROS, reactive oxygen species.

Mitophagy can be part of a developmental programme, such as terminal differentiation of erythrocytes, or be induced by specific conditions, such as hypoxia. In addition, damaged mitochondria are also selectively degraded under normoxic conditions, which prevents accumulation of damaged mitochondria that may cause cell death. Interference in this process may, for example, lead to neuronal degeneration [79]. Selective degradation of depolarized mitochondria involves their recognition by BNIP3 and BNIP3L, which both interact with the autophagosomal membrane protein LC3. BNIP3 and BNIP3L protein levels are upregulated in a HIF1α-dependent manner under hypoxic conditions [80] and during terminal erythroid differentiation [81], suggesting that upregulation of these proteins can trigger mitophagy. BNIP3L prepares mitochondria for autophagic degradation by controlling mitochondrial localization of the E3 ubiquitin ligase Parkin upon depolarization of mitochondria [82]. Translocation of Parkin also depends on the PTEN-induced putative kinase PINK1. PINK1 is imported into all mitochondria where it is maintained at low levels by degradation. If mitochondrial function is impaired, PINK1 is stabilized and accumulates on the OMM of depolarized mitochondria to recruit Parkin [83]. Parkin ubiquitinates mitochondrial proteins, including VDAC1, that serve as a recognition mark for the autophagic machinery [83,84]. Ubiquitinated mitochondrial proteins are bound by p62 (also known as sequestosome 1), which is a multi-functional protein that also interacts with LC3 and thereby promotes association with autophagosomal membranes (figure 2) [85]. Other ubiquitin-binding proteins, such as HDAC6 and NBR1, may also be involved in this process [86,87]. Much of our understanding of the molecular mechanisms of mitophagy has come from studies with CCCP, a mitochondrial uncoupling agent that acutely affects the entire population of mitochondria. Studying mitophagy under more physiological conditions is hard (reviewed in [4]). However, it is reassuring that novel mass spectrometry approaches do support a role for PINK1 and Parkin in mitophagy *in vivo* in *Drosophila* [88].

As a specialized form of autophagy, mitophagy requires suppression of mTORC1 activity and activation of Ulk1 [47]. As indicated above, BNIP3 and BNIP3L prevent mTORC1 activation by directly binding to and inactivating Rheb [48]. Whether p62 also has a role in mTORC1 inactivation is less clear. Upon amino acid stimulation, p62 activates mTORC1 by interacting with Raptor and the Rag GTPases at lysosomes [89]. Translocation of p62 from lysosomes to mitochondria may thus lead to inhibition of mTOR. However, p62 has multiple interaction partners and the relation of p62 and mTORC1 in the process of autophagy is complex [46].

A recent paper shows increased mitophagy when mammalian cells are shifted from glucose-containing medium to glucose-free medium supplemented with glutamine [90]. This change in medium results in a shift from glycolysis to OXPHOS without an alteration of mitochondrial mass, which is accompanied by a selective increase in mitochondrial protein turnover. When the mTOR activator Rheb is transiently overexpressed, a decrease in mitochondrial mass is seen in both types of medium. This is accompanied by an increase in autophagy markers, including BNIP3L, and induces maturation of LC3 that localizes to mitochondria. Also, Rheb is detected at the OMM and can be co-immunoprecipitated with LC3 and BNIP3L. Together, these data show that increased OXPHOS results in a Rheb/BNIP3L-mediated mitochondrial renewal that prevents accumulation of damaged mitochondria. As expected, Rheb stimulates mTORC1, indicating that inhibition of mTORC1 is not a prerequisite for mitophagy under all circumstances. This is consistent with findings in a number of tumour cell lines where high levels of autophagy are found in the presence of active mTOR signalling [91]. A possible explanation may be that under nutrient-limiting conditions ROS are generated that directly activate autophagy via Atg4-mediated processing of LC3 [92].

## 5. Feedback of mitochondrial function on mammalian target of rapamycin complex 1 activity

Homoeostasis depends on feedback systems that allow for balanced adaptive changes. This is also seen in the case of mTOR and mitochondrial function. Under favourable conditions, mTOR promotes mitochondrial biogenesis via transcriptional regulation. However, the increase in mitochondrial mass should be adjusted to nutrient availability in order to prevent malfunctioning of mitochondria. Likewise, starvation responses should lead to a diminished number of mitochondria, but complete removal of mitochondria would be detrimental to a cell. Signalling pathways that coordinate these processes are only partially elucidated and may affect mTOR itself or other elements of the mTOR pathway. Below we briefly discuss recent insights into two mechanisms that involve mitochondrial signalling to mTORC1, namely ROS and the UPR^mt^.

### 5.1. Mammalian target of rapamycin complex 1 activity is regulated by reactive oxygen species

Mitochondrial respiration is a major source for generation of ROS by complexes I, II and III in the mitochondrial electron transport chain (mETC) [93]. The effects of ROS on proteins include oxidation of cysteines and carbonylation. The cellular effects of ROS are complex with dose and cell-type-dependent aspects. Low levels of ROS in a cell can stimulate growth factor-mediated signalling, whereas higher levels induce cell cycle arrest or cell death. Enhanced ROS production is seen under various conditions and context-dependent mechanisms are used to downregulate ROS itself and/or ROS production [94]. Although many effects of ROS on mTORC1 activity are cell-type dependent, there are indications that the effect of ROS on mTORC1 is concentration dependent. Activity of mTORC1 is induced by low levels of ROS, while mild and high levels inhibit mTORC1 activity [95]. ROS can activate mTORC1 via oxidation of cysteine groups. This is seen following treatment of cells with compounds such as phenylarsene oxide (PAO), that specifically induce disulfide bonds. Reducing agents have the opposite effect [96]. ROS and agents like PAO may either oxidize cysteine groups in the mTORC1 complex itself [96] or act at upstream levels, for example the TSC1/2 complex [97]. Mechanisms by which mild and high levels of ROS inactivate mTORC1 are stimulus and cell-type dependent. ROS, generated by mitochondrial depolarization with CCCP, inhibit mTORC1 in an *N*-acetylcysteine-dependent manner, suggesting an involvement of cysteine oxidation [82]. Recently, astrin has been implicated as a mediator of oxidative stress-induced mTORC1 inhibition. Oxidative stress induced by arsenite but also other stresses, for example heat shock, enhance the association of astrin with raptor and consequently diminish the amount of mTOR-bound Raptor [98]. The astrin–raptor complex translocates to stress granules, which are non-membrane-bound cytoplasmic compartments [99]. This results in a decreased mTORC1 activity and can have an anti-apoptotic effect in cancer cells. Reactivation of mTORC1 after resolution of stress can occur by the release of Raptor from stress granules. The dual specificity kinase DYRK3 both helps in dissolving stress granules and, in addition, has a more direct role in mTORC1 activation via phosphorylation of PRAS40 [100]. Most probably, different stresses induce stress granule assembly via distinct molecular mechanisms. How ROS induce stress granules is not exactly known, but it may involve halted translation via phosphorylation of the initiation factor eIF2. Indeed, indications for such a scenario have been documented in *Caenorhabditis elegans* (see below).

It should be noted that various other mechanisms have been reported that cells employ to inhibit mTORC1 after ROS challenge. First, ROS activate stress-activated kinases, for example JNK, that inactivate PI3K signalling via inhibitory phosphorylation of IRS1 [101]. Second, ROS can activate AMPK. ROS can do so via mitochondrial depolarization, which increases levels of AMP. Alternatively, this can occur in an AMP-independent manner [95,102], which, for example, takes place under hypoxic conditions [103].

The inactivation of mTORC1 by ROS represents a feedback mechanism, because it can result in a reduction of the number of mitochondria via mitophagy, and thus prevent further increases in ROS formation [82]. In a similar regulatory mechanism in yeast, nitrogen starvation promotes ROS-induced mitophagy, keeping the number of mitochondria to a minimum to meet energy requirements and simultaneously prevent the production of excess ROS [104].

It should be kept in mind that the inactivation of mTORC1 by ROS is only part of an integral programme by which cells prevent excessive damage. Enhanced ROS production is also counteracted by the antioxidant pathway consisting of JNK and FOXO that leads to upregulation of manganese superoxide dismutase (MnSOD), which converts H_2_O_2_ to H_2_O and O_2_, [105]. Another important transcription factor is NRF2, which controls antioxidant genes such as γ-glutamyl-cysteine ligase, involved in glutathione production. In unstressed cells, NRF2 is degraded following ubiquitination by the E3 ligase KEAP1. Oxidative stress modifies KEAP1 leading to stabilization of NRF2 and its nuclear accumulation [106]. Interestingly, mTORC1 can stimulate NRF2 via direct phosphorylation as a feedback mechanism to protect cells against acute oxidative stress [107].

### 5.2. Mitochondrial unfolded protein response and mammalian target of rapamycin complex 1

Various mitochondrial stress situations can lead to accumulation of misfolded proteins in mitochondria, which triggers the UPR^mt^. The UPR is a well-characterized stress response in eukaryotic cells that relies on molecular chaperones. These chaperones prevent aggregation and promote efficient (re)folding and assembly of newly synthesized and stress denatured proteins. Furthermore, they can also assist in the degradation of irreversibly misfolded or misassembled proteins (reviewed in [6]). A number of genes involved have been identified in an RNAi screen for suppressors of the UPR^mt^ in *C. elegans* [108–111]. The results suggest a model in which proteolysis of mitochondrial proteins and export of the resulting degradation products induce nuclear accumulation of a transcriptional complex consisting of UBL-5 and DVE-1. In addition, diminished import of a basic leucine zipper transcription factor, named ATFS-1, into mitochondria allows ATFS-1 to transfer to the nucleus [112]. Together, these proteins enhance transcription of mitochondrial chaperones and restore protein folding. Interestingly, the RNAi screen also identified the orthologue of Rheb. The exact function of Rheb in this pathway is not clear and initially Rheb and TOR were suggested to act as negative regulators of the nuclear distribution of DVE-1/UBL-5 upon mitochondrial stress [110]. More recent data indicate that repression of cytosolic translation by inhibition of Rheb or mTOR may prevent the induction of mitochondrial stress [108]. This is based on the finding that ROS generated as a consequence of mitochondrial stress slows translation via phosphorylation of the eukaryotic translation factor eIF2α. This decreased translation results in a concomitant diminished requirement for protein folding via a reduction of the import of mETC components [108]. Thus, ROS-mediated inhibition of translation via eIF2α acts in parallel to the UPR^mt^ to protect cells against mitochondrial dysfunction. It is probable that inhibition of mTOR acts in a similar fashion and by inhibition of translation lowers the UPR^mt^.

The mammalian UPR^mt^ appears to operate in a fashion similar to the one described for *C. elegans*, although fewer components of this signalling pathway have been described. The transcription factor that has been identified as a crucial element is a bZIP protein, CHOP, that heterodimerizes with C/EBPβ [113]. Together, these proteins drive the upregulation of mitochondrial stress response proteins, such as the chaperonin Hsp60 and the MM protease ClpP [113,114]. In addition, a separate stress response in the IMS acts to increase CHOP expression [115]. IMS stress also results in ROS production but does not cause a reduction in Δ*ψ*_m_. ROS activate PKB, which in turn activates the oestrogen receptor ERα leading to upregulation of the IMS protease Htra2 and the transcription factor NRF1.

Even though the signalling components of the mammalian UPR^mt^ are incompletely characterized, the importance of UPR^mt^ for health is apparent. The UPR^mt^ resulting from a nuclear–mitochondrial protein imbalance (i.e. the stoichiometric balance between nuclear and mitochondrial-encoded proteins) promotes longevity in mice and *C. elegans* [116]*.* The nuclear–mitochondrial protein imbalance can result from mutation of mitochondrial ribosomal genes or structural components of the ETC, but can also be induced by various drugs. For example, induction of UPR^mt^ occurs following an increase in cytoplasmic nicotinamide adenine dinucleotide levels in *C. elegans*, where it promotes longevity [117]. The fact that UPR^mt^-dependent longevity can be seen in long-lived mutants from the insulin pathway is indicative of separate pathways. However, rapamycin treatment of worms was found to induce UPR^mt^ and the resulting longevity depends on genes functioning in the UPR^mt^. These findings are reminiscent of studies in *Drosophila*, where a link between longevity and translational control of certain nuclear-encoded mitochondrial proteins is documented. Here, dietary restriction results in diminished TOR activity and upregulation of 4E-BP. This specifically enhances translation of complexes I and IV proteins that are important in mediating the lifespan extension [118]. It will be interesting to see whether inducing UPR^mt^ or modulation of mitochondrial quality by other means can be employed to promote healthy ageing.

## 6. Conclusion and perspective

**Figure 3.**
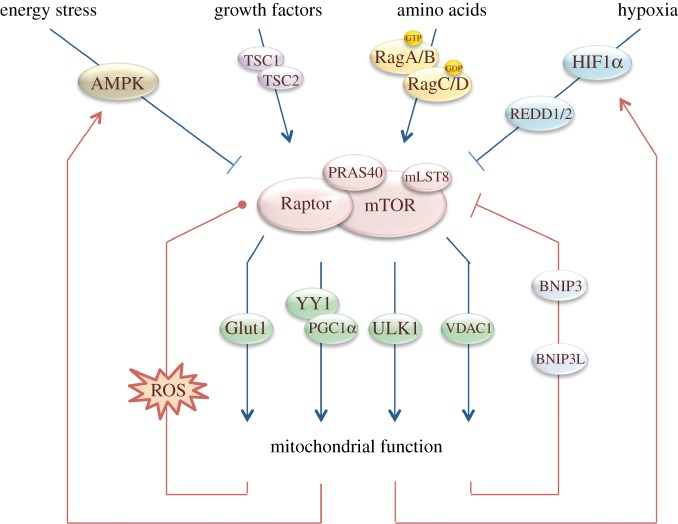
Integrated regulation of mTORC1 activity and mitochondrial function. Growth factors stimulate mTORC1 activity via the inactivation of TSC1/TSC2 complex. Amino acids stimulate mTORC1 activity via the Rag GTPases. Hypoxia inhibits mTORC1 activity via HIF1α stabilization and subsequent REDD1/2 transcription. Energy stress inhibits mTORC1 activity via AMPK. mTORC1 activity regulates mitochondrial homoeostasis via four targets; Glut1, YY1/PGC-1α, Ulk1 and VDAC1. Mitochondrial function regulates mTORC1 by feedback mechanisms. Mitochondrial function regulates HIF1α, AMPK, BNIP3(L) and ROS, which all impact on mTORC1 activity. Low levels of ROS stimulate mTORC1 activity and mild to high levels of ROS inhibit mTORC1 activity. This dual regulation is indicated with a dot. Arrows indicate stimulatory effects. Cross bars indicate inhibitory effects.

Defective mitochondrial homoeostasis can result in tissue-specific effects, eventually causing cancer, neurodegenerative diseases, for example Parkinson's disease, or muscle syndromes, for example dystonia [79]. In this review, we describe how mTOR contributes to mitochondrial homoeostasis (figure 3). mTOR can stimulate mitochondrial biogenesis and function. Simultaneously, mTOR is subjected to the various outputs of mitochondria, including ATP, ROS and metabolic intermediates. In all cases, mTOR functions in complex, cell-type-specific networks with numerous feedback systems. Recent developments reveal that Rheb, the well-known activator of mTOR, functions independently in the control of mitochondrial turnover [90]. Astrin has been identified as a mediator of ROS-induced translocation of Raptor to stress granules [98], while DYRK3 was shown to function in the reactivation following stress [100]. Classical forward genetic screens in *C. elegans* are continuing to identify novel proteins that function in mitochondrial homoeostasis by either acting in the UPR^mt^ or modifying this response [108]. With rapid technical developments in metabolic profiling, cell culture systems using patient-derived cells and techniques to visualize ROS inside cells we will hopefully reach a more complete understanding of the reciprocal signalling of mTORC1 and mitochondria and be able to offer new prospects for treatment of mitochondria-related diseases.
